# Evolution of short cognitive test performance in stroke patients with vascular cognitive impairment and vascular dementia: Baseline evaluation and follow-up

**DOI:** 10.1590/1980-57642016dn11-040007

**Published:** 2017

**Authors:** Nilton Custodio, Rosa Montesinos, David Lira, Eder Herrera-Perez, Yadira Bardales, Lucia Valeriano-Lorenzo

**Affiliations:** 1 Unidad de Diagnóstico de Deterioro Cognitivo y Prevención de Demencia. Instituto Peruano de Neurociencias. Lima, Peru.; 2 Servicio de Neurología. Instituto Peruano de Neurociencias. Lima, Peru.; 3 Servicio de Medicina de Rehabilitación. Instituto Peruano de Neurociencias. Lima, Peru.; 4 Unidad de Diseño y Elaboración de Proyectos de Investigación. Instituto Nacional de Salud del Niño. Lima, Peru;; 5 GESID. Lima, Peru.; 6 Unidad de Geriatría. Instituto Peruano de Neurociencias. Lima, Peru.; 7 Unidad de Neuropsicología. Instituto Peruano de Neurociencias. Lima. Peru.

**Keywords:** vascular dementia, vascular cognitive impairment, Addenbrooke's Cognitive Examination, INECO Frontal Screening, cohort, demencia vascular, comprometimento cognitivo vascular, Exame Cognitivo de Addenbrooke, INECO Frontal Screening, coorte

## Abstract

There is limited evidence about the progression of cognitive performance during the post-stroke stage.

**Objective:**

To assess the evolution of cognitive performance in stroke patients without vascular cognitive impairment (VCI), patients with vascular mild cognitive impairment (MCI), and patients with vascular dementia (VD).

**Methods:**

A prospective cohort of stroke outpatients from two secondary medical centers in Lima, Peru was studied. We performed standardized evaluations at definitive diagnosis (baseline evaluation), and control follow-ups at 6 and 12 months, including a battery of short cognitive tests: Clinical Dementia Rating (CDR), Addenbrooke's Cognitive Examination (ACE), and INECO Frontal Screening (IFS).

**Results:**

152 outpatients completed the follow-up, showing progressive increase in mean score on the CDR(0.34 to 0.46), contrary to the pattern observed on the ACE and IFS (78.18 to 76.48 and 23.63 to 22.24). The box plot for the CDR test showed that VCI patients had progressive worsening (0.79 to 0.16). Conversely, this trend was not observed in subjects without VCI. The box plot for the ACE and IFS showed that, for the majority of the differentiated stroke types, both non-VCI and VCI patients had progressive worsening.

**Conclusion:**

According to both ACE and IFS results during a 1-year follow-up, the cognitive performance of stroke patients worsened, a trend which was particularly consistent in infarction-type stroke patients.

## RESUMO.

Há evidências limitadas sobre a progressão do desempenho cognitivo durante o estágio pós- acidente vascular cerebral (AVC).

**Objetivo:**

Avaliar a evolução do desempenho cognitivo em pacientes com AVC sem comprometimento cognitivo vascular (SCCV), pacientes com comprometimento cognitivo leve vascular (CCL-V) e pacientes com demência vascular (DV).

**Métodos:**

Coorte prospectivo de pacientes ambulatoriais com AVC de dois centros médicos secundários de Lima, Peru. Realizamos avaliações padronizadas no diagnóstico definitivo (avaliação inicial) e controles aos 6 e 12 meses depois, incluindo um conjunto de testes cognitivos breves: Clinical Dementia Rating (CDR), Addenbrooke's Cognitive Examination (ACE), and INECO Frontal Screening (IFS).

**Resultados:**

Completaram o estudo 152 pacientes ambulatoriais, mostrando que as médias de pontuação da CDR apresentaram aumento progressivo (0,34 a 0,46), contrariamente ao observado com ACE e IFS (78,18 a 76,48 e 23,63 a 22,24). A soma das caixas do teste CDR mostrou que os pacientes com comprometimento cognitivo vascular (CCL-V e DV) apresentaram piora progressiva (0,79 a 0,16). Por outro lado, em indivíduos SCCV, essa tendência não foi observada. O gráfico box-plot para ACE e IFS mostrou que, para a maioria dos tipos de AVC observados, tanto os pacientes SCCV como aqueles com CCV apresentaram piora progressiva.

**Conclusão:**

De acordo com os resultados de ACE e IFS durante o acompanhamento de 1 ano, o desempenho cognitivo em pacientes com AVC piora, o que é particularmente consistente em pacientes com AVC tipo infarto.

## INTRODUCTION

Cerebrovascular diseases (CVD) (such as infarction, hemorrhage, large artery disease, cardiac embolism, small-vessel disease, among others) cause cerebral vascular injury (CVI) that can lead to vascular cognitive impairment (VCI), a syndrome in which cognitive impairment can be attributed to vascular disorders.^1^ Thus, there are two main clinical forms of VCI, including VCI caused by recent and symptomatic stroke (post-stroke VCI) and VCI caused by “covert” CVI (VCI without recent stroke), such as silent brain infarction, hemorrhage, and white matter lesions, detectable only on neuroimaging or at autopsy.^2^

According to its severity, VCI may be subdivided into either mild cognitive impairment (MCI) or dementia.^3^^,^ 4 MCI is defined based on the presence of acquired cognitive problems, representing a decline from a previous level of functioning, accompanied by objective evidence of impairment on cognitive testing but with essentially preserved activities of daily living. In dementia, impairments are sufficiently severe to affect activities of daily living.

Some autopsy studies have shown that small clinically unrecognized infarctions are very common and associated with dementia,^11^^-^ 14 consequently, many cases of both vascular or mixed dementia are misclassified as Alzheimer's disease (AD).^2^ Despite this, vascular dementia (VD) is the second-most-common type of dementia,^5^ accounting for up to 17 million cases worldwide with annual costs of up to 200 billion U.S. dollars.^6^ Thus, dementia caused by vascular disease as well as AD, i.e. mixed dementia, is the most common cause of later life dementia.^7^

Despite its importance for public health, there is limited literature about the progression of cognitive performance in patients during the post-stroke stage. Thus, the aim of this study was to assess the evolution, during one year, of cognitive performance in stroke patients without VCI, patients with vascular MCI (VCI without dementia), and patients with VD.

## METHODS

### Study design.

A prospective, 12-month cohort study was conducted between July 2011 and June 2017 involving stroke outpatients from two secondary medical centers in Lima, Peru (the “Clínica Internacional” and the “Instituto Peruano de Neurociencias”).

### Selection of patients.

Eligible patients were individuals over 55 years old, with Spanish as their native language, at least 4 years of education, and at least 30 days post-cerebral stroke. We excluded patients with any neurodegenerative disease (mild cognitive impairment, AD, Parkinson's disease, fronto-temporal dementia (FTD), or Lewy body dementia), dementia secondary to medical causes, history of psychiatric disorders (depression or schizophrenia), aphasia or consciousness impairment that hindered communication ability, and with structural and/or functional deficits (motor, visual or auditory) because these can affect performance on the neuropsychological tests. During the follow-up, we excluded patients who suffered additional cerebral stroke events, did not complete the evaluations/tests of the study, or dropped out of the study.

Because depression and apathy are potential confounding variables, at baseline and during follow-up, we excluded probable cases, according to their results on screening tests: the Beck Depression Inventory–Second version (BDI-II).^8^^-^ 10 and Apathy scale (AS),^11^ respectively. Therefore, patients with a BDI-II score ≥ 14 and AS score ≥ 14 were excluded.

### Definitions

#### Stroke.

According to the criteria of the WHO MONICA Project Investigators, we defined cerebral stroke as the existence of rapidly evolving clinical signs that are a consequence of focal/global disturbance of brain function, with symptoms lasting more than one hour and consistent findings on brain CT scan or magnetic resonance imaging, in the context of absence of apparent non-vascular causes.^12^ Thus, this study enrolled patients with ischemic stroke, and patients with other CVD/CVI such as transient ischemic attack (atherothrombotic infarction, lacunar infarction, cardio embolic, and non-determined embolism) and hemorrhagic events (parenchymal and subarachnoid hemorrhage).

#### Vascular Cognitive Impairment (VCI).

Cognitive syndrome caused by CVD/CVI, in which the manifestations of cognitive deficits exceed those of normal aging. Eventually, VCI progresses to affect daily living activities and social/occupational functioning, i.e. VD.^4^^,^ 13

### Procedures.

This cohort was constructed by use of a dementia protocol providing a standardized approach to demented outpatients. Applying this protocol, the evolution of the disease was assessed at definitive diagnosis (baseline evaluation), and at 6 and 12 months later (first and second control follow-ups).

#### Stroke diagnosis.

Because in the setting of a stroke event functional impairments may also result from its motor and sensory consequences,^2^ our team of neurologists rigorously evaluated each case to determine the degree to which functional impairments were directly attributed to cognitive impairment or were secondary to the consequences of stroke.

Based on neuroimaging and neuropathology studies, the evidence shows that progressive accumulation of ‘covert' CVD/CVI (i.e. clinically silent CVD/CVI) in the absence of evident stroke is sufficient to cause clinically relevant cognitive impairment.^14^^,^ 15 Thus, the absence of a clinical history of stroke does not exclude VCI. For this reason, our sample comprised only stroke cases confirmed by brain CT scan or magnetic resonance imaging, regardless of the history of apparent stroke.

#### Baseline evaluation.

Following our study protocol, a complete clinical evaluation was performed, including: 1) interview for sociodemographic information (age, sex, education) and history; 2) physical examination by neurologists to perform a complete neurological evaluation and measure weight, height, and waist circumference; 3) clinical examination by neurologists to apply the modified Hachinski index, modified Barthel index (MBI), National Institutes of Health Stroke Scale (NIHSS), and neuropsychological short tests; 4) neuropsychological assessment by neuropsychologists; and 5) assessment of standard laboratory tests, including measurement of levels of hemoglobin, glucose, serum urea, serum creatinine, glutamic-oxalacetic and gluta­mic-pyruvic transaminases, albumin, globulin, vitamin B12, folic acid, thyroid profile (free T3 and T4, and ultra-sensitive TSH) and serum electrolytes (sodium, potassium and chloride), and serologic test for syphilis (VDRL) and HIV screening test (ELISA).

The cognitive battery for neuropsychological assessment encompassed the following tests: Rey Auditory Verbal Learning Test (Rey, 1941), Logical Memory – Subtest of the Wechsler Memory Scale-Revised (Wechsler, 1997), Trail Making Test A and B (Partington et. al., 1949), Rey-Osterrieth Complex Figure Test (Rey, 1941), Boston Naming Test (Kaplan et. al., 1983), Wisconsin Card Sorting Test (Nelson, 1976), Letter-Number and Cubes Test – Subtests of the Wechsler Adult Intelligent Scale III (Wechsler, 1997), and copy of drawing (Strub and Black, 1988). The neuropsychiatric and functional assessments were performed using the Neuropsychiatric Inventory and the Alzheimer's Disease Cooperative Study – Activities of Daily Living inventory (ADCS-ADL), respectively.

We applied the following neuropsychological short test: 1) Clinical Dementia Rating (CDR);^16^ 2) Addenbrooke's Cognitive Examination (ACE);^17^^,^ 18 and 3) INECO Frontal Screening (IFS).^19^^,^ 20

#### Diagnosis.

Based on the diagnostic criteria of the American Heart Association/American Stroke Association,^4^ the team of neurologists and neuropsychologists reached a consensus for the following diagnostic categories: cognitively normal patients without vascular cognitive impairment (non-VCI) and patients with VCI. The patients with VCI were differentiated into: 1) vascular MCI, according to the criteria of the Canadian Study of Health and Aging;^21^ and 2) VD, according to the criteria of the Stroke – Association Internationale pour la Recherche et l'Enseignement en Neurosciences (NINDS–AIREN).^22^

In contrast with older definitions of dementia, newer definitions of VD do not require evidence of memory impairment or impairments in more than one cognitive domain.^3^^,^ 4 ^,^ 23 This allows a diagnosis of VD to be established in the not uncommon setting where a patient with VCI has severely impaired executive function but preserved memory and other cognitive domains.

#### Follow-up.

We carried out control follow-ups at 6 and 12 months after diagnosis to re-evaluate neuropsychological status using the same tests.

#### Data analysis.

Descriptive statistics were applied to all variables, including measures of frequency distribution for qualitative variables, as well as central tendency (mean or median) and of statistical variability (standard deviation or minimum and maximum values) for quantitative variables. Box plots were used to map the progression of performance on the cognitive short tests, differentiated by etiology and by neuropsychological diagnosis. For exploratory purposes, progression of BDI-II was also assessed.

Analyses were performed using the statistical software package STATA, version 12.0 (StataCorp LP College Station, TX, USA). The information on patients was anonymized and de-identified prior to analysis.

#### Ethics statement.

Written informed consent was obtained from all participants (or their relative in the case of dependent patients) whose clinical records were used for this study. The study protocol was approved by the ethics committee of the Universidad San Martín de Porres of Lima, Peru.

## RESULTS

**Figure 1:**
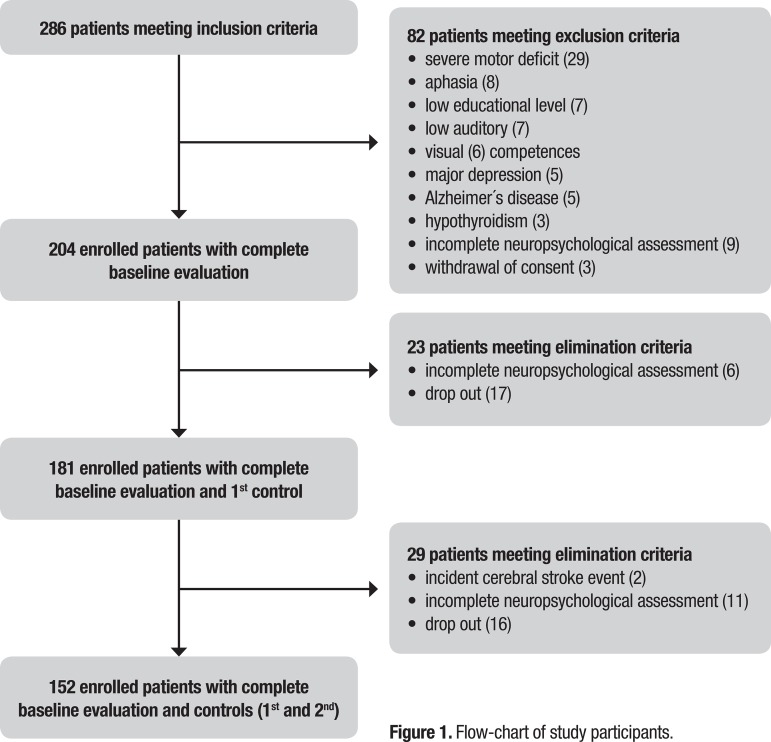
Flow-chart of study participants.

In the present study, 152 outpatients completed the follow-up (Figure 1). The rate of loss during the entire follow-up was 25.5%. The cohort that completed follow-up comprised a sample that was 50% female with mean age of 68.82 years, 11.69 years of formal education, 93.32 on the Barthel scale, and 1.72 on the NIH Stroke Scale/Score. These variables had similar values for all three evaluation timepoints (baseline, and first and second control follow-ups) (Table 1).

**Table 1:** Demographic characteristics, neuropsychological test scores and neuropsychological diagnosis in stroke patients from Lima-Peru, according to evaluation timepoint.

		Evaluation timepoint		
		Baseline evaluation (n = 204)	1^st^ Control (n = 181)	2^nd^ Control (n = 152)
Sex: female		105 (51.47%)	91 (50.28%)	76 (50%)
Age, years*		69.12 (4.65)	68.78 (4.59)	68.82 (4.75)
Education, years*		11.57 (3.37)	11.69 (3.39)	11.69 (3.51)
Barthel scale, score*		93.09 (4.10)	93.37 (4.02)	93.32 (4.21)
NIH Stroke Scale/Score, score*		1.74 (1.41)	1.67 (1.38)	1.72 (1.39)
Neuropsychological diagnosis	Without VCI	124 (60.78%)	111 (61.30%)	77 (50.80%)
	VCI without dementia	61 (29.90%)	48 (26.50%)	46 (30.20%)
	Vascular dementia	19 (9.31%)	22 (12.20%)	29 (19%)
CDR, score*		0.34 (0.51)	0.39 (0.58)	0.46 (0.63)
ACE, score*		78.18 (10.18)	77.24 (10.07)	76.48 (9.98)
IFS, score*		23.63 (4.23)	23.22 (4.24)	22.24 (4.67)
BDI-II, score*		5.22 (2.84)	5.23 (1.76)	5.58 (1.27)
Apathy scale, score*		8.81 (3.11)	8.05 (2.28)	7.05 (2.24)

VCI: Vascular cognitive impairment; CDR: Clinical Dementia Rating; ACE: Addenbrooke's Cognitive Examination; IFS: INECO Frontal Screening; BDI-II: Beck Depression Inventory – Second version;

* Data expressed as mean (standard deviation).

**Supplementary table:** Demographic characteristics and neuropsychological test scores at baseline evaluation in patients with CVI from Lima-Peru, according to neuropsychological diagnosis.

	Neuropsychological diagnosis		
	Non-VCI (n = 124)	VCI without dementia (n = 61)	Vascular dementia (n = 19)
Sex: female	60 (48.39 %)	34 (55.74%)	11 (51.47%)
Age, years*	69.07 (4.53)	68.21 (4.57)	72.31 (4.46)
Education, years*	11.91 (3.47)	11.09 (3.20)	10.84 (3.13)
Barthel scale, score*	93.37 (4.33)	93.20 (3.26)	90.73 (4.42)
NIH Stroke Scale/Score, score*	1.77 (1.41)	1.51 (1.30)	2.32 (1.63)
CDR, score*	0.48 (0.15)	0.55 (0.15)	1.58 (0.61)
ACE, score*	84.79 (6.86)	69.32 (3.36)	63.52 (4.10)
IFS, score*	26.49 (1.52)	20.57 (1.82)	14.78 (2.18)
BDI-II, score*	4.37 (2.61)	5.97 (2.63)	8.32 (2.11)
Apathy scale, score*	9.18 (3.40)	8.26 (2.60)	8.16 (2.17)

VCI: Vascular cognitive impairment; CDR: Clinical Dementia Rating; ACE: Addenbrooke's Cognitive Examination; IFS: INECO Frontal Screening; BDI-II: Beck Depression Inventory – Second version;

* Data expressed as mean (standard deviation).

During the follow-up, the proportion of VCI (without dementia) in the sample showed a progressive increase. Thus, 30.20% and 19.00% of the patients evaluated at the 2^nd^ control follow-up had VCI without dementia and vascular dementia, respectively. Regarding the neuropsychological short test, the data showed a progressive increase in average scores on the CDR and BDI-II, and a progressive decline in average scores on the ACE and IFS (Table 1).

**Figure 2:**
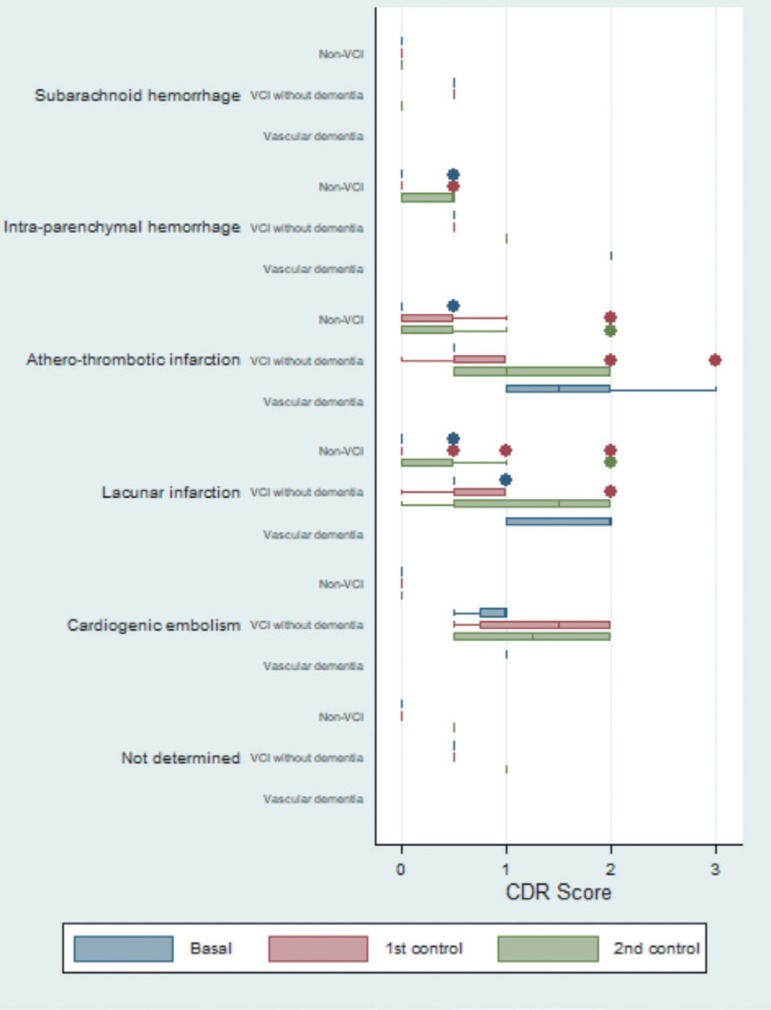
Box plot to graph of the evolution of the scores of Clinical Dementia Rating (CDR), differentiated by etiology and by neuropsychological diagnosis.

The box plot for the CDR test showed a difference in scores by etiology. The patients with atherothrombotic infarction had the highest values. For the majority of the differentiated etiologies, the data showed that VCI patients had a progressive worsening relative to baseline status. Conversely, this trend was not observed in subjects without VCI (Figure 2).

**Figure 3:**
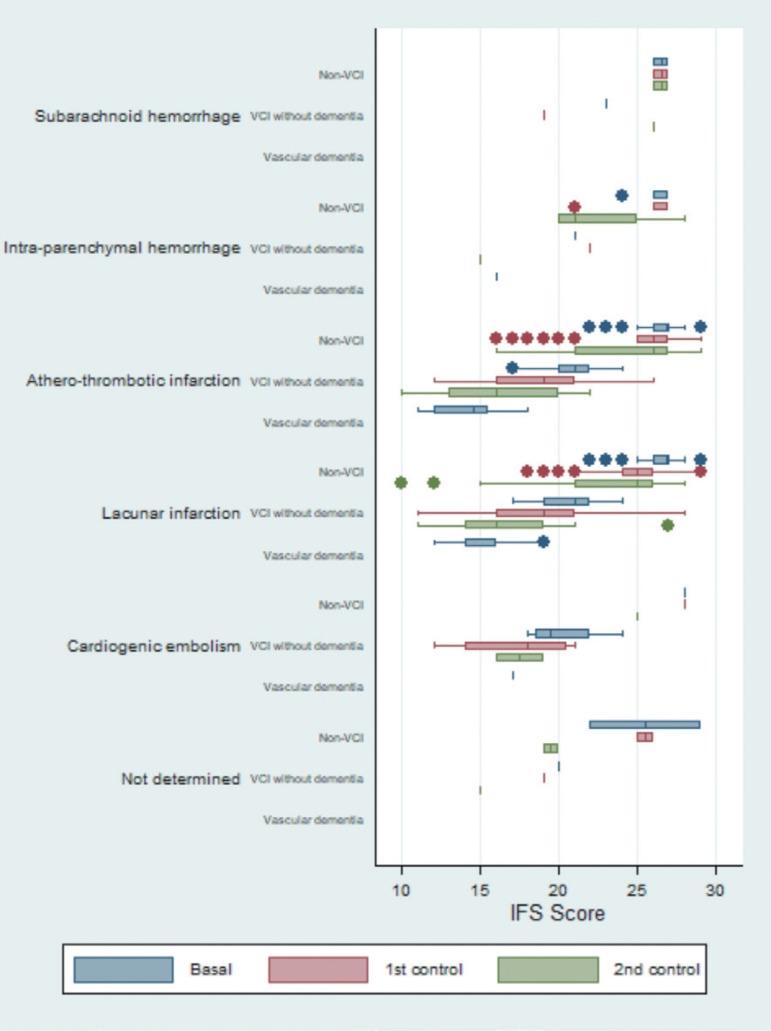
Box plot to graph of the evolution of the scores of Addenbrooke's Cognitive Examination (ACE), differentiated by etiology and by neuropsychological diagnosis.

**Figure 4:**
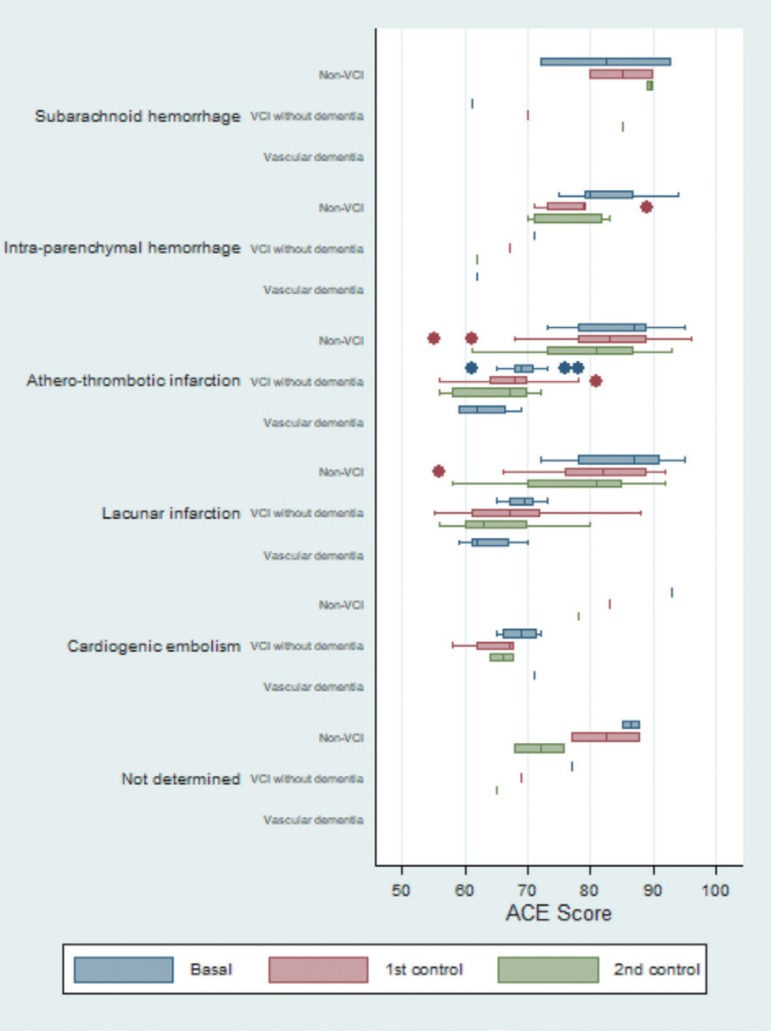
Box plot to graph of the evolution of the scores of INECO Frontal Screening (IFS), differentiated by etiology and by neuropsychological diagnosis.

The box plot for the ACE test showed similar progression patterns among the different etiologies, particularly for atherothrombotic infarction and lacunar infarction. Patients with atherothrombotic infarction and lacunar infarction had the lowest values. For the majority of the differentiated etiologies, the data showed that both non-VCI and VCI patients had progressive worsening relative to baseline status. The only exception was subarachnoid hemorrhage (Figure 3). Similar results were observed for the IFS test (Figure 4).

**Figure 5:**
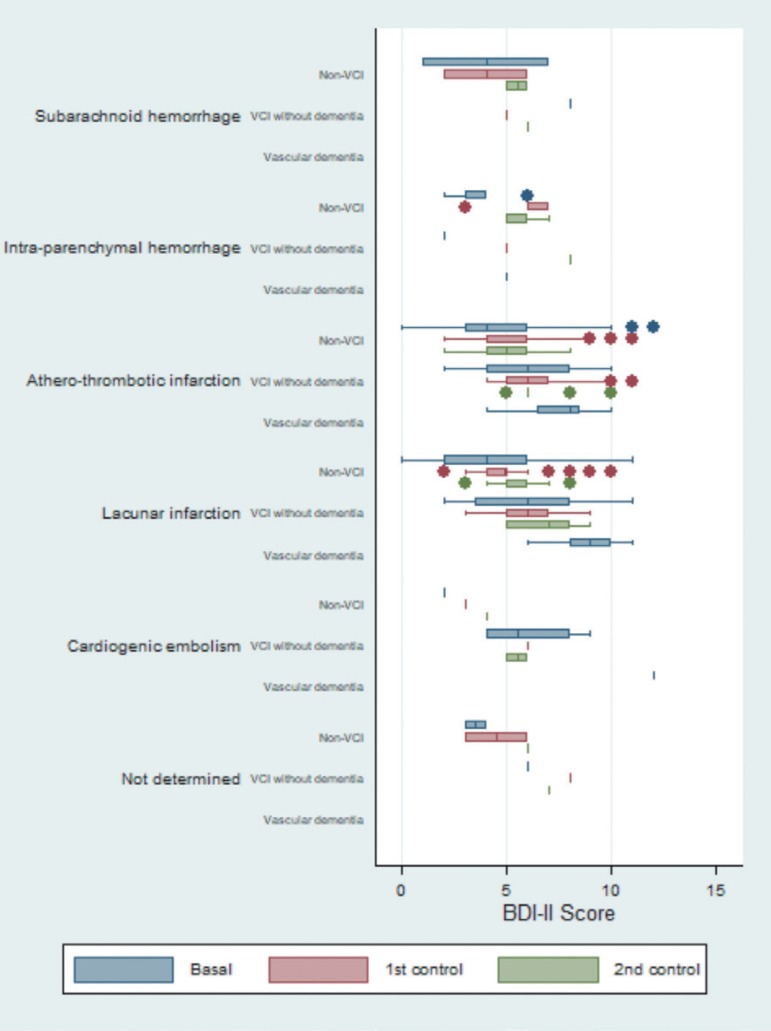
Box plot to graph of the evolution of the scores of Beck Depression Inventory – Second version (BDI-II), differentiated by etiology and by neuropsychological diagnosis.

The box plot for BDI-II showed similar progression among the different etiologies, particularly for atherothrombotic infarction and lacunar infarction. Patients with atherothrombotic infarction and lacunar infarction had the highest values. For the majority of the differentiated etiologies, the data showed that both non-VCI and VCI patients had progressive worsening relative to baseline status. The only exception was subarachnoid hemorrhage (Figure 5).

Regarding expected progression patterns, some apparent inconsistences were observed in the group of patients with subarachnoid hemorrhage and VCI without dementia (Figures 2-5).

## DISCUSSION

This study showed that, based on the majority of applied short tests, cognitive performance worsened as time progressed in stroke patients with VCI, but this was also observed in some stroke patients without VCI. This pattern was particularly evident in patients with medical history of atherothrombotic infarction and lacunar infarction. These results are consistent with previous prospective longitudinal studies, which reported a progressive increase in the cognitive deterioration rate.^24^^,^ 25

The clinical and cognitive profile of VCI differs from other causes of cognitive impairment and dementia.^3^ In our study, we found that infarction-type stroke patients had similar cognitive profiles on both ACE and IFS tests. Because of the small size of these groups, we were unable to identify a clear cognitive pattern in the case of other etiologies. Future studies should focus on patients with subarachnoid hemorrhage, intra-parenchymal hemorrhage, and cardiogenic embolism.

In general, there was an acceptable inter-individual variation on the cognitive performances across all the short tests. For infarction-type stroke patients, variability on the ACE test appeared to be less than on the IFS test. However, this variability progressively reduced only on the IFS test. This finding should be further evaluated by pooled longitudinal studies to establish both the reliability and consistency of these short cognitive tests over time.

Regarding mean scores at baseline evaluation, the ACE performance of stroke patients was higher than that previously reported in Peruvian patients with AD (67.00; SD = 5.26) and FTD (76.61 SD = 4.90). Similarly, IFS performance was higher than that previously reported in Peruvian patients with AD (20.02; SD = 2.10) and FTD (14.55; SD = 2.16).^20^ These findings suggest that the ACE could be a useful tool for discriminating between VCI/VD and AD, and IFS for discriminating between VCI/VD and FTD.

The MMSE is the most used short cognitive test but has shown shortcomings for detecting dysexecutive abnormalities, which are characteristic of VCI disorders. In this context, The ACE and IFS do not share these drawbacks and are potentially useful tests for both cognitively assessing and following up stroke patients.^3^^,^ 26 Our team has validated these short cognitive tests in Peruvian samples of AD and FTD patients.^18^^,^ 20 Thus, we recommended validating both the ACE and IFS tests in Latin American patients for discriminating VCI in stroke patients.

However, there is wide inter-individual variation in the manifestations of vascular brain injury along with overlap with other non-VCI causes of dementia, such that clinical signs and symptoms may suggest the diagnosis of VCI but radiological or pathological confirmation of vascular brain injury is usually needed. In this review, VCI diagnostic criteria, epidemiology, and clinical manifestations were reviewed.

Limitations. Since comorbid pathologies such as AD are commonly identified and pathological diagnosis is needed to rule out the existence of underlying neurodegenerative disorder,^27^ it is unclear whether our sample comprised only patients with pure VCI.

Because some VCI lesions are undetectable by the current technique of brain MR (such as microinfarcts, which are only visible under light microscopy), the frequency of VCI may have been underestimated.^26^ Thus, our study is biased toward VCI cases secondary to evident CVD/CVI.

In conclusion, in the stroke patients, cognitive performance worsened during the 1-year follow-up, even in patients without VCI. This decline is consistent and progressive, particularly in infarction-type stroke patients (atherothrombotic infarction and lacunar infarction), who showed similar cognitive profiles on both ACE and IFS tests. We believe that both the ACE and IFS can be good tests for discriminating VCI/VD from other neurodegenerative disorders, such as AD and FTD. However, rigorous studies assessing this hypothesis should be conducted.
